# Who’s cooking tonight? A time-use study of coupled adults in Toronto, Canada

**DOI:** 10.1177/0961463X221100696

**Published:** 2022-05-21

**Authors:** Bochu Liu, Michael J Widener, Lindsey G Smith, Steven Farber, Dionne Gesink, Leia M Minaker, Zachary Patterson, Kristian Larsen, Jason Gilliland

**Affiliations:** Department of Geography and Planning, 7938 University of Toronto—St George, Toronto, ON, Canada; Department of Human Geography, 33530 University of Toronto Scarborough, Toronto, ON, Canada; Dalla Lana School of Public Health, 7938 University of Toronto, Toronto, ON, Canada; School of Planning, 8430 University of Waterloo, Waterloo, ON, Canada; Concordia Institute of Information Systems Engineering, 5618 Concordia University, Montreal, QC, Canada; Department of Geography and Planning, 7938 University of Toronto––St George, Toronto, ON, Canada; CAREX Canada, Faculty of Health Sciences, 1763 Simon Fraser University, Burnaby, BC, Canada; Department of Geography and Environment, Department of Paediatrics, Department of Epidemiology and Biostatistics, School of Health Studies, 6221 Western University, London, ON, Canada

**Keywords:** food-related housework, food preparation and cooking, gendered household labor division, time allocation, cohabiting partners

## Abstract

Understanding how coupled adults arrange food-related labor in relation to their daily time allocation is of great importance because different arrangements may have implications for diet-related health and gender equity. Studies from the time-use perspective argue that daily activities such as work, caregiving, and non-food-related housework can potentially compete for time with foodwork. However, studies in this regard are mostly centered on individual-level analyses. They fail to consider cohabiting partners’ time spent on foodwork and non-food-related activities, a factor that could be helpful in explaining how coupled partners decide to allocate time to food activities. Using 108 daily time-use logs from seventeen opposite-gender couples living in Toronto, Canada, this paper examines how male and female partners’ time spent on non-food-related activities impact the total amount of time spent on foodwork by coupled adults and the difference in time spent on foodwork between coupled women and men. Results show that both male and female partners took a higher portion of foodwork when their partner worked longer. When men worked for additional time, the couple-level duration of foodwork decreased. Without a significant impact on the gender difference in foodwork duration, women’s increased caregiving duration was associated with a reduction of total time spent on foodwork by couples. An increase in caregiving and non-food-related chores by men was associated with an increased difference in duration of foodwork between women and men, which helped secure a constant total amount of foodwork at the couple level. These behavioral variations between men and women demonstrate the gender differences in one’s responsiveness to the change of partners’ non-food-related tasks. The associations found among non-food-related activities and foodwork are suggestive of a need to account for partners’ time allocation when studying the time-use dynamics of foodwork and other daily activities.

## Introduction

Food-related housework (foodwork for short) includes a range of activities that occur inside the home, like preparation, cooking, and cleaning up. Engagement in these activities is closely linked to the consumption of food, and in turn influences individuals’ diet-related health outcomes (Wolfson and Bleich, 2015). Despite a decline of home cooking practices in the past decades, foodwork still occupies a large portion of the time spent on routine housework and is often shared in partnered households (Plessz and Étilé, 2019). The division of labor can take various forms, often resulting in one partner devoting more time to foodwork than the other (Klünder and Meier-Gräwe, 2017; Kolpashnikova and Kan, 2021; Lake et al., 2006; Taillie, 2018). Among opposite-gender couples, there can be a tendency towards gendered labor division with more of the foodwork being undertaken by women (Bianchi et al., 2000; Klünder and Meier-Gräwe, 2017). Research on same-sex households showed that, compared to heterosexual couples, household labor is more equally shared, especially among lesbian couples because of more liberal attitudes toward gender roles (Goldberg et al., 2012; Smart et al., 2017). While the dynamics between partners can vary based on many different factors, with gender being one, this paper will focus on the dynamics present in opposite-gender partners due to a lack of same-sex partner representation in our dataset (see Goldberg (2013) and Brewster (2017) for more on division of housework in same-sex couples). Because of this, the remainder of the introduction will concentrate on how foodwork is arranged in heterosexual couples, which has profound implications for gender equity and diet-related health.

Women’s disproportional share of foodwork in opposite-gender couples has raised concerns around gender inequity given the high regularity of food chores and the large portion of time these activities take. The uneven assignment of foodwork between genders can further exacerbate the constraints that inhibit women from participating in leisure and other activities related to their well-being (Clifford Astbury et al., 2020). To address these concerns, researchers have endeavored to understand the factors associated with the unequal division of labor, with large duration of paid work and commuting trips being identified as a major contributor (Ettema and van der Lippe, 2009). A typical argument these studies make is that when facing binding time constraints, partners have to coordinate their activity arrangements to fulfill their personal and household needs, which is commonly shown by a specialization of work-related and household labor between partners (Ettema and van der Lippe, 2009). Though partners’ time allocation has been recognized as a substantial factor shaping coordination of household labor (Ettema et al., 2007), whether and how the difference in time spent on foodwork between coupled adults is conditioned on their work and other non-food-related tasks remains underexplored.

Related to the gendered division of foodwork, the total amount of time allocated to foodwork can be variable at the household level. This indicator is closely related to the types of meals people consume and is therefore a critical determinant for their diet-related health outcomes (Mills et al., 2017). Homemade meals are usually more nutritious than foods prepared at other places such as take-away and prepackaged meals (Celnik et al., 2012; Wolfson and Bleich, 2015), but the consumption of home prepared meals is usually time-consuming as it requires participation in cooking and other food-related chores. When residents experience binding time constraints such as long hours of work and irregular working schedules, they are inclined to substitute home food preparation with quicker options (Devine et al., 2009; Jabs and Devine, 2006; Liu et al., 2021). The extensive availability of ready-to-eat meal options further facilitates people to shorten or skip food-provisioning processes (Virudachalam et al., 2014). These individual-level analyses of how a person alters food-provisioning behaviors in response to time constraints, however, misses the ways cohabiting partners’ time allocation comes into play in the arrangement of foodwork. By conducting a couple-level analysis, this study will examine whether and how the total amount of time spent on foodwork in a household is related to coupled adults’ time allocated to non-food-related activities.

This paper will focus on heterosexual couples and explore how they navigate food-related responsibilities in the context of non-food-related daily activities. Specifically, we will examine how partners’ time allocated to non-food-related activities impacts both the total amount of time spent on foodwork at the couple level and the difference in time spent on foodwork between coupled women and men using a sample of opposite-gender couples living in Toronto, Canada. While men’s and women’s time spent on household labor has converged in recent decades in Canada and other Western countries, a stubborn gender asymmetry still remains (Guppy et al., 2019; Milkie et al., 2021; Sayer, 2010). An examination of coupled partners’ time devoted to foodwork will shed light on gendered divisions of household labor in the kitchen (Widener et al., 2021).

## Literature review

Past research on food-related activities and couples can be grouped into two areas: theoretical considerations and empirical patterns of division of food-related household labor; and associations between partners’ daily time allocation and arrangement of food-related household labor.

### Theoretical considerations and empirical patterns of division of food-related household labor

Relative resources, time availability, and gender are three major theoretical perspectives for explaining division of household labor (Horne et al., 2018; Mandel et al., 2020; Shelton and John, 1996). Grounded in social exchange theory in sociology, the perspective of relative resources posits that specialization of housework reflects a rational decision by coupled partners according to their relative socio-economic statuses and power differentials (Coverman, 1985; Lundberg and Pollak, 1996; Moreno-Colom, 2017). In this view, the spouse with higher economic status (e.g., higher earnings from employment) has the power to negotiate a labor delegation in which he or she does a lower share of housework while the economically dependent partner is expected to do more (Becker, 1981; Coverman, 1985; Grunow et al., 2012). This proposition is supported by the observations of a lower share of household labor for women with a higher economic status (Ettema and van der Lippe, 2009; Presser, 1994).

In addition to the economic resources, time availability has been recognized as a critical factor impacting division of household labor. Activities of high priority and activities highly fixed in space or time, like paid work and caregiving, affect how much time is available for housework (Coverman, 1985; Cullen and Godson, 1975). Following this rationale, coupled partners are more likely to assign the most of housework to one partner when encountering more extensive time constraints (e.g., long hours of work) (Ettema and van der Lippe, 2009). Across nine Western industrialized countries including the US, the UK, and Canada, more time in employment decreases women’s and men’s duration of housework because of less available time, albeit stronger effects are observed for women (Sayer, 2010). Nevertheless, the perspectives of relative resources and time availability assume a gender-neutral process in which either male or female partner can use their resources and constraints to negotiate their share of household labor (Sayer, 2010). The assumption of the gender-neutral process is challenged by counterintuitive observations that a male partner whose female partner earns more than he does undertakes less housework compared to other men (Bittman et al., 2003). In more extreme situations where men experience forced unemployment, the more women take on outside employment, the less their male partners involve in housework (Legerski and Cornwall, 2010). These counter examples suggest that gender comes into play in determining the division of domestic labor.

Gender theorists propose the concept of “doing gender” to understand the production of gender through what one does, and does recurrently in interactions with others (West and Zimmerman, 1987). Berk (1985) argued that doing housework and childcare and the division of such labor produces gender. “Femaleness” is confirmed by doing housework and “maleness” by eschewing it (Berk, 1985; Mandel et al., 2020; Treas and Tai, 2012). When men are involved in housework, they are inclined to focus on “masculine” tasks like the upkeep of home, yard, and automobiles, while women undertake the more time-intensive and routine “feminine” housework including cooking and cleaning (Treas, 2008). The gendered strategies working parents employ often result in women doing “the second shift” of housework and childcare (Hochschild, 1989). As a natural consequence of gender specialization in household tasks, a reduced overlap in the skill sets and motivations of the partners will make the male partner less ready to substitute the tasks the female partner routinely performs and thus reinforce the gendered specialization patterns (Treas, 2008).

Studies based on nationally representative time-use diary surveys showed a general decrease in women’s housework, with some corresponding increases in men’s housework across highly industrialized countries in Europe and North America (Bianchi et al., 2006; Kan et al., 2011; Sayer, 2010). This narrowing gender gap was also observed in time spent on foodwork, and was argued to be primarily driven by increasing numbers of women working full time and increased participation in housework by men with high socio-economic profiles (Klünder and Meier-Gräwe, 2017; Taillie, 2018). Nonetheless, women still bear a higher load of routine household labor including meal preparation (Kan et al., 2011). Studies revealed that women, in general, take on more food-related tasks than men in terms of duration (Guppy et al., 2019; Kolpashnikova and Kan, 2021; Moreno-Colom, 2017), participation rate (Klünder and Meier-Gräwe, 2017, 2018; Taillie, 2018), and perceived responsibilities (Lake et al., 2006) in the US, Canada, and western European countries. On top of the gender asymmetry of household labor that still remains, men’s increase in housework slowed down in recent decades (Sayer, 2010; Sullivan et al., 2018), causing a debate on whether the gender gap in housework will keep converging. Proponents of a stalled gender convergence argued that a new cultural frame of egalitarian essentialism combined with a rhetoric of choice and equality fueled a return to conservative gender role expectations (Cotter et al., 2011). However, other scholars projected that, despite its slow pace, the gender gap in domestic labor will continue to converge considering generational changes (Sullivan et al., 2018). By analyzing up-to-date time-use data of coupled partners, this study will provide evidence of the current patterns of household labor division with a focus on foodwork.

### Associations between partners’ daily time allocation and arrangement of foodwork

Couched in theoretical perspectives of time availability, studies have shown that limited time is associated with reduced food-provisioning activities by resorting to various coping strategies (Jabs and Devine, 2006). For instance, when a person reported having limited time resources, especially when they had long working hours and irregular working schedules, they were more likely to substitute time-intensive at-home food provision activities with quicker alternatives (e.g., ready-to-eat prepackaged meals) (Alonso-Domínguez et al., 2020; Devine et al., 2009; Jabs and Devine, 2006). These findings align with the consideration that activities of high priority or fixed in location or time (e.g., work) can influence the participation and duration of more flexible activities (e.g., maintenance activities) (Cullen and Godson, 1975; Fransen et al., 2018; Hägerstrand, 1970). Nevertheless, the important potential confounder of a partner’s time use was missed in those studies.

The arrangement of food-related household tasks is contingent on partners’ time allocation to work. In the interview portion of a study on adults in their thirties from the UK, not working or having a partner working full time were cited as reasons for being responsible for food shopping and cooking, and more flexible working schedules than a partner’s was reported to make it more convenient for an adult to shop for food (Lake et al., 2006). A German time-use survey showed that women’s time allocation outcomes were more subject to household employment arrangements, while men’s contribution to foodwork is insusceptible to women’s work. Women in the households consisting of a non-working woman and a male primary earner reported higher amount of time spent on meal preparation than women from dual-earner households and households comprising a male primary earner and a female additional earner. In contrast, men’s duration of meal preparation was approximately the same among the three configurations of households (Klünder and Meier-Gräwe, 2018). These findings echo observations from earlier work (Baxter, 2002) and suggest that men tend to be less responsive to changes in women’s work duration. Whether gendered responses to partners’ work duration exist for households from other regions and of different ethnic backgrounds is worth exploring.

Studies on within-household time allocation adopt holistic conceptualizations of between-partner interactions in not only work, but also non-work activities (Zhang et al., 2005; Zhang and Fujiwara, 2006). For example, using time-use data of a Chinese sample of coupled partners, Cao and Chai (2007) detected negative relationships between a person’s work duration and their duration of maintenance activities as well as positive associations between a person’s work duration and his/her partner’s duration of maintenance activities. This study, however, did not find any significant associations between partners’ maintenance activities. With a more detailed classification of maintenance activities, Ettema et al. (2007) found a negative association between men’s duration of out-of-home household tasks and women’s duration of in-home household tasks, as well as a negative association between women’s duration of out-of-home personal business and men’s duration of in-home household tasks. These associations suggest a need to account for possible linkages between the partner’s time allocation for both work and non-work activities and a person’s engagement in household labor.

Since previous studies pursuing interpersonal interactions of time use combine foodwork into coarser activity categories of maintenance or housework, it remains unclear whether and to what extent a partner’s time use specifically interacts with a person’s foodwork. Different behavioral responses to partners' time use can lead to varied arrangements of food chores in a household. Considering the situation where one partner faces binding temporal constraints and then reduces duration of food-related tasks, if the other partner is unable or unwilling to devote more time to foodwork, a reduction in the total household duration of food-related chores is expected. If the other partner performs food-related tasks for a longer time in such situations, the total time spent on food-related chores at the household level may not change much thanks to the adjusted division of foodwork.

### Posited associations between time spent on non-food-related daily activities and assignment of foodwork among coupled adults

This study formulates two hypotheses regarding the associations between non-food-related activities and foodwork.

Posited association 1: Men’s paid work, caregiving, and non-food-related housework in relation to coupled adults’ foodwork:

When men increase their duration of paid work, caregiving, or non-food-related housework, it is expected that an increased *difference in duration of foodwork between women and men* and an unchanged *total household duration of foodwork* will be observed.

Posited association 2: Women’s paid work, caregiving, and non-food-related housework in relation to coupled adults’ foodwork:

When women increase their duration of paid work, caregiving, or non-food-related housework, it is expected that a reduced *difference in duration of foodwork between women and men* and a reduced *total household duration of foodwork* will be observed.

Informed by the theories and past findings on how time availability and gender influence the division of household labor, we assume that activities usually of higher fixity and priority will compete for time resources with foodwork and men will be less responsive to women’s increased time consumed by work, caregiving, and non-food-related housework. When women spend increased amounts of time on non-food-related activities, their time spent on foodwork will decrease but their male partners will not spend more time on foodwork to compensate for this reduction. This will thus lead to a narrowed difference in duration of foodwork between women and men and a reduction of the total household duration of foodwork. By contrast, women will spend more time on foodwork to compensate for the reduction in men’s time spent on foodwork, when men allocate more time to non-food-related activities. Such behavioral responses will result in an increased difference in duration of foodwork between women and men, and an unchanged total household duration of foodwork. Examining whether these associations exist will help disentangle when and how non-food-related activity durations of coupled adults impact the total amount of household foodwork and the gender difference in duration of foodwork.

## Methods

### Data

**Figure 1.**
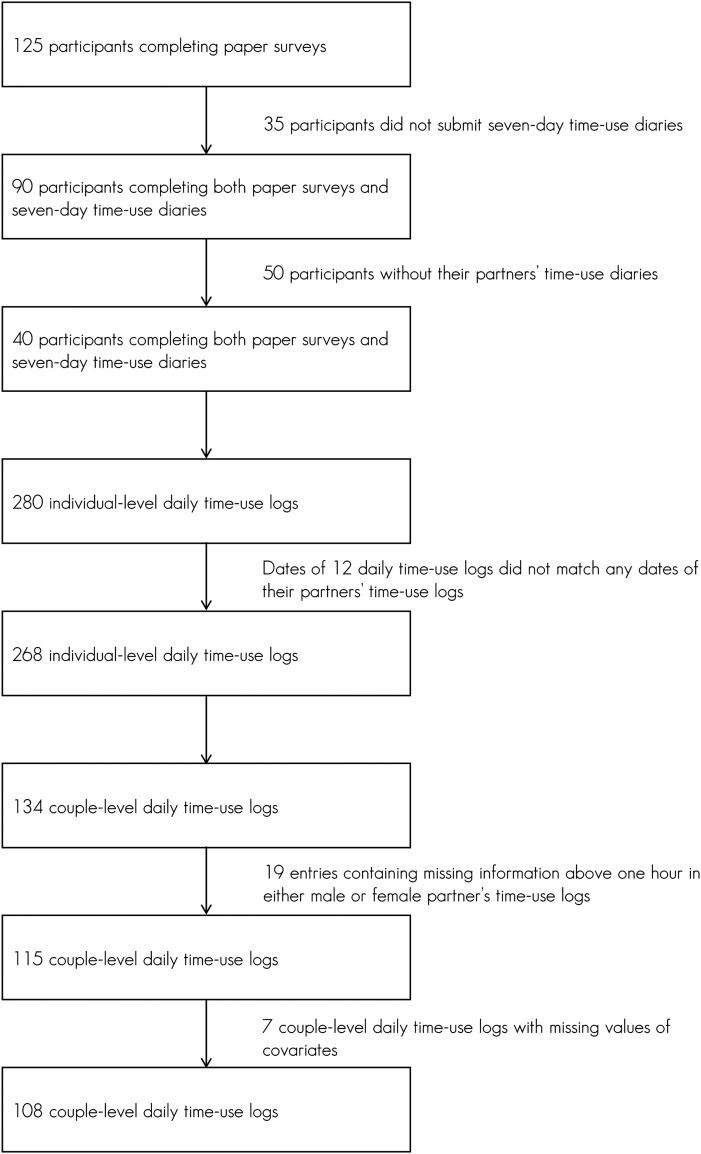
Selection of the analytical sample.

We used data collected in Toronto, Ontario, Canada in March 2019 from the Food Activities, Socio-economics, Time use, and Transportation (FASTT) Study. Individuals between 18 and 65 years old were recruited, along with their partners, through random intercept interviews conducted by the research team in the low-to-moderate-income neighborhoods of Parkdale, Rexdale, and West Hill within the city of Toronto (see Smith et al., 2022 for detailed description of the data collection; see Appendix A for detailed description of the three study neighborhoods). The portion of the FASTT dataset used in this study consisted of a paper survey about socio-demographics and food behaviors and a 7-day time-use diary. The FASTT study collected information about co-residing household members by inviting all the adult household members from the residence of the initially recruited eligible individual to participate. This collection approach rendered it possible to acquire daily time-use logs of coupled adults, which will be of interest for this paper. Among the 125 participants completing the paper survey, 90 submitted time-use diaries for seven complete days. In the orientation session, participants were asked if their partners also participated. If their partner did participate, then their survey packages were coded with the same prefix as their partner’s. Self-reported residential locations of both partners were crosschecked to ensure cohabitation. Only coupled adults were used for this analysis. Fifty participants were dropped due to a lack of their partners’ time-use information. Forty participants with partners’ time-use diaries consisted of 280 daily entries. 12 daily entries were dropped that did not align with their partners’ entries (due to a few cases where coupled partners did not start tracking time use from the same day). Thus, a subset of 268 individual days with corresponding daily time-use diaries from a partner was constructed and converted to 134 couple-days where entries of paired adults with the same date were combined. To ensure the accuracy of duration measures, 19 couple-days where either partner had time slots of missing information above 1 hour were not included. An additional seven daily entries with missing values of the covariates were discarded, resulting in a final analytical dataset that contains 108 couple-days from 17 couples (see Figure 1).

With couple-day as the unit of analysis, we derived durations of activities of interest from coupled partners’ time-use diaries. The format of the time-use diary was adapted from the Harmonised European Time Use Survey (HETUS) (European Union, 2019), which asked participants what they were doing, where they were, and who they were within 144 10-min intervals on a given day. No predefined activity categories were provided in the form and the participants were asked to describe what they were doing in their own words. This was later coded by research assistants to identify activity categories that closely align with the activity classification scheme used in the General Social Survey of Canada Time Use Activity Cycle 29 (Statistics Canada, 2019).

To align our analysis with the study hypotheses, we derived five activity categories of work, caregiving, non-food-related housework (hereafter, other housework), foodwork, and recreation. Work in this study refers to paid work and training activities. Caregiving includes care of children under 18 years and adults in the household. The specific activities of this category are personal care of care receivers, getting ready for school or daycare, supervising or helping with homework, accompanying to or from school and other activity locations the care receivers need to go, and accompanying during activities in which care receivers participate.

The category of household chores used in the original classification scheme is dichotomized into food-related chores and non-food-related ones (Statistics Canada, 2019). Foodwork comprises unpacking groceries, meal preparation or cooking, snack preparation, and dishwashing or cleaning up after a meal. All the other chores at home, such as laundry and house cleaning, are classified as other housework. Recreation activity in this study is used as an umbrella term for socializing, active sports and events, active leisure (e.g., playing video games), and passive leisure (e.g., watching TV). Informed by previous findings highlighting varied within-household interactions for in-home and out-of-home activities of the same type (Ettema et al., 2007), this study distinguishes out-of-home activities from in-home ones for all the aforementioned activity categories (except housework) based on the self-reported locational information in the time-use diaries. Table 1 shows the descriptive statistics of the duration variables. In the FASTT sample, women took on a larger share of foodwork than their male partners.

**Table 1.** Descriptive statistics of daily activity duration for men (*n* = 54) and women (*n* = 54) (unit: minute).

Activity categories	Men (*n* = 54 couple-days)^a^			Women (*n* = 54 couple-days)^a^		
		95% confidence interval			95% confidence interval	
	Mean [mins/day]	Lower bound [mins/day]	Upper bound [mins/day]	Mean [mins/day]	Lower bound [mins/day]	Upper bound [mins/day]
Work						
At-home^b^	38.70	19.01	58.40	5.28	−2.22	12.78
Out-of-home^b^	248.43	202.66	294.19	110.00	79.07	140.93
Housework						
Food-related^b^	50.74	38.78	62.70	141.94	125.16	158.73
Non-food-related^b^	15.09	8.67	21.52	45.09	33.22	56.97
Caregiving						
At-home^b^	31.94	20.20	43.69	81.76	62.69	100.83
Out-of-home^b^	24.44	13.47	35.42	39.54	28.42	50.66
Recreation						
At-home	146.76	122.00	171.52	135.56	113.51	157.60
Out-of-home	20.00	10.95	29.05	11.02	4.04	17.99

Note: ^a^The daily observations included both weekdays and weekend days.

^b^A significant difference in men’s and women’s activity duration was found in paired Wilcoxon signed-rank test at a significance level of 0.05. Non-parametric tests for difference in means were used to account for the non-normality of activity duration variables.

### Variables and analytical approach

The daily observations of the same couples can be used to unveil the dynamic relationships of coupled adults’ time allocations across days, which required the use of analytical approaches that can handle both day-level and couple-level variables. This study employed multi-level linear regressions with random effects to explore the associations between partners’ time spent on non-food-related activities and arrangement of foodwork.

The first outcome variable of interest is the total duration of foodwork, defined as the sum of time spent on food-related housework by coupled partners on a day. The second outcome variable is the difference between partnered adults in duration of foodwork, and is measured by subtracting the male partner’s time spent on foodwork from that of their female partner’s because the time women spend on food chores is typically greater (Table 2). Summary statistics of the two outcome variables are shown in Table 2.

**Table 2.** Summary statistics for outcome variables (unit: minute).

	Mean	SD	Minimum	P25	Median	P75	Maximum
Total within-household duration of daily foodwork	192.69	92.33	20.00	120.00	185.00	260.00	440.00
Difference in duration of daily foodwork between women and men	91.20	123.89	−200.00	10.00	100.00	162.50	400.00

To test the hypotheses pertaining to the associations between non-food-related activities and food chores, the regression models include duration of work, caregiving, other housework, and recreation as the explanatory variables. Work, caregiving, and recreation activities were further classified into at-home and out-of-home activities to account for more fixed constraints incurred by the latter. The models controlled for a dummy variable representing suburban neighborhoods to capture potential variations in foodwork between couples residing in varying residential environments. In this study, Parkdale was categorized as an urban neighborhood, while the other two neighborhoods were regarded as suburban. Possible variation between weekdays and weekend days in outcomes was considered by controlling for a dummy variable indicating if the day was a weekend day.

A man’s and a woman’s age were controlled for as age is associated with the capability of doing housework (Coltrane, 2000). Household income in the past 12 months was controlled for given its relevance to the ability to outsource foodwork (e.g., eating out frequently and buying domestic services) (Cohen, 1998; Legerski and Cornwall, 2010). The models also adjusted for ethnicity. Considering both partners of 10 couples (58.8% of the total number of couples) and one partner of an additional couple were of South Asian descent, a dummy variable of “South Asian couple” was created where a true value was given if at least one partner was of South Asian descent. Presence of other household members cohabiting with the coupled adults may relate to the total duration of foodwork and how those tasks are allocated (Jabs et al., 2007; Mills et al., 2017; Ta et al., 2016), so the models adjust for whether coupled adults were co-residing with children under 18, adults aged 18–64, or older adults above 64. Having young children has been shown to be linked to more traditional housework arrangements (Davis and Greenstein, 2004; Presser, 1994). Other research has shown that cohabiting with an elder mother or an adult child could reduce the likelihood women turn to their male partners for help with household labor (Treas, 2008). Additionally, one’s perceptions and capabilities of cooking is reported to impact who does food preparation and cooking (Lake et al., 2006; Mills et al., 2017). This is accounted for by using responses to a question that asked to what extent the participant enjoys cooking. The distribution of the aforementioned socio-demographic variables are shown in Table 3. The analytical sample is mostly young and middle-aged couples with children from suburban neighborhoods. More than half of the couples had at least one South Asian partner and household income less than $60,000. A higher proportion of women reported that they enjoy cooking.

**Table 3.** Distributions of socio-demographic variables of coupled adults (*n* = 17).

Variables	Count of all couples (*n* = 17) *n* (%)^a^	Variables	Count of all couples (*n* = 17) *n* (%)^a^
Age		Cooking enjoyed by male partner^f^	
30–39	9 (52.9%)	Not at all	4 (23.5%)
40–49	4 (23.5%)	Sometimes, or under some circumstances	8 (47.1%)
50–59	2 (11.8%)	Yes, but I would prefer not to cook	0 (0.0%)
60–69	2 (11.8%)	Very much	5 (29.4%)
Household income in the past 12 months^b^		Cooking enjoyed by female partner	
Less than $30,000	6 (35.3%)	Not at all	0 (0.0%)
$30,000 to less than $60,000	3 (17.6%)	Sometimes, or under some circumstances	4 (23.5%)
$60,000 to less than $90,000^c^	5 (29.4%)	Yes, but I would prefer not to cook	2 (11.8%)
$90,000 to less than $120,000	2 (11.8%)	Very much	11 (64.7%)
$120,000 and above	1 (5.9%)	Living with children under 18	
South Asian couple^d^		No	3 (17.6%)
No	6 (35.3%)	Yes	14 (82.4%)
Yes	11 (64.7%)	Living with adults between 18 and 64 years old	
Residential neighborhood		No	11 (64.7%)
Urban	3 (17.6%)	Yes	6 (35.3%)
Suburban^e^	14 (82.4%)	Living with older adults above 64 years old	
		No	14 (82.4%)
		Yes	3 (17.6%)

Notes: ^a^Percentage for column is shown within parentheses.

^b^The unit is Canadian dollar.

^c^One couple of which the male partner reported $60,000 to less than $90,000 while the female partner reported $30,000 to less than $60,000 was grouped into $60,000 to less than $90,000 in this table. In the regression analysis, the average household income for the two partners was used.

^d^Participants were asked about their racial and cultural backgrounds and the majority of the sample identified themselves as South Asian. A couple is categorized as South Asian if at least one of them is of South Asian descent.

^e^Rexdale and West Hill are grouped as suburban residential neighborhoods.

^f^Whether respondents enjoyed cooking is quantified by response to the question “Do you enjoy cooking?"

The mixed linear regressions can account for the multi-level structure of the data where daily observations are nested within households by incorporating random effects. The random intercepts capture the couple-level variations unexplained by the fixed terms, so that the estimated coefficients of daily duration variables can reflect the associations between non-food-related activities and food chores at the daily level (e.g., how the total duration varies across days with the daily changes of one partner’s caregiving duration). The regression analyses are operationalized using lme4 package in R (Bates et al., 2015; R Core Team, 2020).

## Results

### Partners’ non-food-related activity duration and total daily duration of foodwork

The estimation results from the multi-level linear regressions with random intercepts are shown in Table 4. Duration of work and caregiving activities was significantly associated with total household duration of food-related chores. Men’s duration of out-of-home work was related to a reduced amount of time spent on food-related chores by coupled adults, suggesting that men’s work activities compete for time resources with foodwork. Women’s out-of-home work duration however was not significantly associated with total duration of household food chores. The difference in the effects of men’s and women’s out-of-home work activities suggests that the total amount of time spent on foodwork did not change significantly when women worked for a longer time outside of home. This result echoes with the previous findings of women’s invariant time spent on foodwork regardless of women’s work statuses (Klünder and Meier-Gräwe, 2017, 2018).

**Table 4.** Estimates of multi-level linear regressions with random effects predicting total daily duration of foodwork and difference in daily duration of foodwork between woman and man (*n* = 108).

	Total daily duration of foodwork			Difference in daily duration of foodwork between woman and man		
Fixed effects						
	Coefficient	95% CI		Coefficient	95% CI	
(Intercept)	489.83*	267.45, 741.04		216.50	−95.09, 565.78	
Man’s duration of at-home work	−0.18	−0.47, −0.05		0.16	−0.09, 0.33	
Woman’s duration of at-home work	0.02	−0.33, 0.32		−0.38**	−0.70, −0.10	
Man’s duration of out-of-home work	−0.10 *	−0.17, 0.00		0.21***	0.10, 0.27	
Woman’s duration of out-of-home work	−0.08	−0.21, 0.05		−0.15**	−0.33, −0.05	
Man’s duration of non-food-related housework	−0.31	−0.75, 0.21		0.65***	0.14, 1.04	
Woman’s duration of non-food-related housework	0.20	−0.08, 0.49		0.10	−0.07, 0.54	
Man’s duration of at-home caregiving	0.07	−0.19, 0.31		−0.13	−0.63, −0.01	
Woman’s duration of at-home caregiving	−0.36 **	−0.61, −0.19		−0.01	−0.37, 0.13	
Man’s duration of out-of-home caregiving	0.06	−0.21, 0.36		0.36 **	−0.06, 0.54	
Woman’s duration of out-of-home caregiving	−0.25	−0.51, 0.00		0.02	−0.12, 0.53	
Man’s duration of at-home recreation	−0.07	−0.20, 0.08		0.01	−0.09, 0.21	
Woman’s duration of at-home recreation	0.03	−0.17, 0.17		−0.01	−0.12, 0.24	
Man’s duration of out-of-home recreation	−0.29	−0.59, 0.05		0.07	−0.18, 0.49	
Woman’s duration of out-of-home recreation	−0.29	−0.70, 0.10		−0.48**	−1.00, −0.19	
Weekend	−29.53	−66.96, 4.82		−2.39	−49.06, 22.30	
Cooking enjoyed by man	−5.44	−22.62, 16.29		3.57	−23.92, 26.70	
Cooking enjoyed by woman	−8.73	−31.71, 14.66		10.47	−16.19, 46.45	
Co-residing with adults aged 18–64	−79.84	−153.41, −34.85		−126.10	−224.51, 20.32	
Co-residing with children under 18	−18.69	−114.98, 56.80		−49.08	−141.78, 19.24	
Co-residing with older adults above 64	102.08*	58.01, 155.54		146.90	84.74, 229.66	
Man’s age	−10.36	−19.14, −4.05		−5.84	−17.31, 2.19	
Woman’s age	8.52	3.16, 15.65		3.32	−3.88, 12.57	
Household income	−13.19	−28.73, 9.75		−30.84	−58.44, −8.71	
South Asian	61.47	14.91, 116.70		81.21	23.87, 157.72	
Inner suburb neighborhood	−40.79	−108.48, 38.42		16.27	−95.91, 101.48	
Random effects						
Groups	Name	Variance	Std. Dev.	Name	Variance	Std. Dev.
Couples	(Intercept)	956.20	30.92	(Intercept)	6320.00	79.50
Residual		4600.60	67.83		3291.00	57.37
Model fit						
Number of days	108			108		
Number of couples	17			17		
Log likelihood	−576.71	(df = 28)		−567.28	(df = 28)	
AIC	1209.43			1190.55		
BIC	1284.53			1265.65		

Note: ***, **, *denote a significance level of 0.01, 0.05, and 0.10, respectively.

Women’s duration of at-home caregiving was significantly associated with a decreased amount of total household duration of food-related chores (Table 4). Given the lack of an association between the duration of women’s out-of-home caregiving activities and the gender difference in duration of food chores (Table 4), it was highly likely that both women and men reduced their duration of foodwork in the sampled households when women increased the duration of caregiving. Additionally, cohabiting with older adults above 64 was associated with a higher duration of foodwork at the couple level (Table 4), which may be related to increased responsibilities of providing meals for household members.

### Partners’ non-food-related activity duration and gender difference in daily duration of foodwork

Both men’s and women’s work duration significantly influenced the difference in duration of foodwork between genders (Table 4). A one-minute increase in women’s duration of at-home work was significantly associated with a decrease of 0.38 min (95% CI: −0.70, −0.10) in the difference in time spent on food chores between women and men. To put this into context, for a subsample of coupled adults residing in Toronto, gender difference in time spent on food chores was expected to decrease by 22.68 min (95% CI: 5.76, 42.06) with a 1-h increase in women’s at-home work duration, holding covariates constant. Duration of women’s out-of-home work was also negatively associated with the gender difference in duration of foodwork, with a 0.15-min (95%: −0.33, −0.05) decrease for every minute increase in women’s duration of out-of-home work. This indicated that when women worked for an additional 60 min, they were expected to reduce the daily gender gap of foodwork by 9.24 min (95% CI: 3.18, 19.56). These results suggested that the division of foodwork became more even when women spent more time on work-related activities. On the contrary, men’s duration of out-of-home work was significantly associated with an increase of 0.21 min (95% CI: 0.10, 0.27) in the difference in foodwork duration between women and men. To put this into context, when men extended their daily work duration by an hour, the gender difference in foodwork duration was expected to increase by 12.54 min (95% CI: 5.94, 16.38), implying that higher work duration of men was likely to enlarge the gender difference in foodwork between women and men.

Men’s daily time spent on other housework (i.e., non-food-related housework) and caregiving activities was related to an expanding gender gap of time spent on foodwork (Table 4). A one-minute increase in men’s duration of other housework was associated with a 0.65-min (95% CI: 0.14, 1.04) increase in difference in foodwork duration between women and men. For every minute increase in men’s duration of out-of-home caregiving, the gender difference was expected to increase by 0.36 min (95% CI: −0.06, 0.54). These positive relationships between men’s time allocated to non-food-related household tasks and difference in women and men’s time spent on foodwork suggested that the division of foodwork became more uneven when men spent more time on non-food-related household labor.

Additionally, a 1-min increase in women’s duration of out-of-home recreational activities was associated with a 0.48-min (95% CI: −1.00, −0.19) decrease in the difference in women’s and men’s duration of foodwork (Table 4), indicating a more even food labor division. This provides an insight into task sharing and trade-offs in time use between partners whereby one compensates for their partner’s participation in recreational activities by taking on more of the foodwork.

## Discussion

The associations observed between non-food-related activities and foodwork from the FASTT dataset had some discrepancies with the posited associations. Both male and female partners took a higher portion of foodwork when their partner worked longer. The expected associations between women’s work duration and foodwork was not supported by the empirical finding, as men spent more time on foodwork when women’s work duration increased and there was no statistically significant decrease in the total time spent on foodwork at the couple level. The posited association pertaining to men’s work duration was not fully supported neither. The total time spent on foodwork decreased when men worked for additional time, despite an increased gender difference in foodwork.

The associations between caregiving or other housework and foodwork were in line with the posited associations. An increase in men’ time spent on caregiving or other housework was associated with an increased gender difference in foodwork duration without any significant change in the total foodwork duration at the couple level. In contrast, given the little change in gender difference, additional time women spent on caregiving was related to a reduction of the total household duration spent on foodwork. These findings were suggestive of persisting gender differences in household roles, as well as gender differences in responsiveness to changes in partners’ time spent on non-food-related tasks.

### Gendered associations between non-food-related activities and foodwork

The results show that the gender difference in duration of foodwork remained almost unchanged when women spent additional time on caregiving, in contrast with a larger gender gap when men took more on caregiving (Table 4). A similar finding was shown in situations in which other housework consumed more time of the male partner. These results suggested that women may still be responsible for foodwork even with increased caregiving responsibilities whilst men could do less foodwork when they did additional non-food-related household tasks. This gender difference may be attributed to the larger amount of time men spend on paid labor, compared to their female partners. In our study, men’s average duration of out-of-home work was 248.43 (95% CI: 202.66, 294.19) minutes, over two times of 110.00 min (95% CI: 79.07, 140.93) that women spent on this activity (Table 1). The gender gap in outside work was even larger for suburban couples, among which men spent 251.49 (95% CI: 200.13, 302.86) minutes while women only allocated 69.77 (95% CI: 41.29, 98.25) minutes on average. The gender specialization in paid work can impact couples’ foodwork arrangement in two ways. On the one hand, long hours men spend on paid work constrain their ability to respond to the demands to do foodwork. On the other hand, fewer hours women spend on employment may leave them more time available for performing household chores, diminishing the demands on their male partners to fulfill these responsibilities (Coverman, 1985). Nevertheless, the gender differences in duration of paid work could be a reflection of gendered expectations related to the division of household labor. Other research has also found that women from Canada generally perform fewer hours of paid work per week on average than men, as they tend to spend more time on housework and childcare (Moyser, 2017).

The gendered associations between non-food-related household tasks and foodwork suggest that traditional gender expectations and the “doing” of gender remain prominent in shaping the ways coupled adults arrange foodwork. Men’s lack of response in foodwork to women’s increased caregiving duration implies that cooking and caring for family members were probably still considered as “women’s work.” According to traditional gender norms, meal preparation and caregiving are intimately tied to the (problematic) female roles of “wife” and “mother” and performing these activities entails a symbolic enactment of gender (Berk, 1985; Kerr and Charles, 1986; Robinson and Milkie, 1998). Coupled partners influenced by conservative gender expectations, which can be heightened in particular cultures and social networks (Barker, 2011), are likely to produce and reaffirm their gender through a division of labor in which women are primarily responsible for routine household labor including cooking and childcare (Treas, 2008). Women who have internalized these gender expectations as their perceived responsibility will be reluctant to ask their male partners to substitute the chores they usually do even when they encounter difficulty juggling work and housekeeping responsibilities (Allen and Hawkins, 1999; Legerski and Cornwall, 2010). Meanwhile, men holding the traditional gender expectations may perceive devoting more time to foodwork unnecessary when women handle additional caregiving tasks. In contrast with men’s absent response, women’s compensation for men’s reduced time in foodwork when men undertook additional caregiving and non-food-related housework suggests that additional contribution to non-food-related household labor can possibly result in men not taking part in certain foodwork.

Despite different responses to partners’ non-food-related household labor by gender, both men’s and women’s time in cooking were responsive to their partners’ work duration. Given that paid work is usually associated with binding time constraints (e.g., working hours) not open for negotiation, coupled adults must accommodate for the changing work duration of either male or female partners. The elasticity of men’s cooking to women’s work also corresponds to the previous finding of working women’s higher propensity than their non-working counterparts to ask their male partners to substitute the housework they routinely did, which was argued to be a result of their male partners’ preparedness to help in terms of skills and motivations (Treas, 2008).

### Implications for gender equity and dietary health

In situations where one person faces extensive time constraints imposed by work and non-food-related household tasks, distributing foodwork to his/her partner and reducing the total amount of foodwork at the couple level become reasonable strategies, which are connected to issues of gender equity and dietary health.

This study shows that women took on a larger share of foodwork than their male partners, which aligns with results from German and US time-use surveys (Klünder and Meier-Gräwe, 2017; Taillie, 2018). More importantly, this study unveils the trade-offs between non-food-related activities and foodwork among coupled men and women. Despite a narrowed gender gap in time spent on cooking in the past decades (Taillie, 2018), the disparities in responsiveness to partners’ changing non-food-related labor between men and women are still indicative of the persistent gendered labor division in which women are primarily responsible for foodwork and other household labor while men’s involvement in these activities is considered by some to be optional. The gendered ways of responding to partners’ time pressures imply that the conservative gender expectations may still inform perceptions and feelings around being a “good” partner in today’s society (Bianchi and Milkie, 2010; Kan et al., 2011; Moreno-Colom, 2017; Treas, 2008; Treas and Drobnic, 2010). Neutralizing the meaning of cooking and caregiving traditionally attached to gender will help encourage men to increase their engagement in these activities and move towards a more equal coordination of household labor (Ettema and van der Lippe, 2009; Grunow et al., 2012). Moreover, in line with previous findings of the equalizing effect of women’s employment on housework time (Fuwa, 2004; Sayer, 2010), the finding that men responded to women’s increased work duration by spending more time on foodwork suggests that supporting women’s employment and increasing their time spent on employment may help effectively equalize the division of foodwork.

This study also observes a few scenarios in which the total time spent on foodwork decreased (e.g., when women took on more care activities). The reduced time spent on foodwork may contribute to skipping meals or substituting at-home meal preparation with quicker alternatives (e.g., prepackaged and take-away foods) commonly associated with unbalanced nutritional intake (Celnik et al., 2012). Our study finds that couples reduced their total duration of foodwork on days when men spent more time on work or women spent more time on care outside of the home. This initial evidence could inform health policymakers and practitioners about the potential targets for social, behavioral, and built environment interventions. Offering job opportunities and childcare services more accessible to coupled adults may be the key to alleviating the time constraints pertaining to work and childcare and increasing time available for food chores (Devine et al., 2009; Venn and Strazdins, 2017).

The small sample size limited the generalizability of the findings. However, the confidence in the analysis of partners’ time allocation is increased by using multi-level models with random effects and controlling for confounders like personal cooking preferences. This study complements past work of national time-use data by providing insights into the ways couples share and coordinate food-related tasks using data collected contemporaneously from both partners. It also distinguishes at-home activities from out-of-home ones and delineates a diverse range of activities that potentially compete for time with foodwork. Large-scale household time-use surveys can be used to derive more robust associations between various non-food-related activities and foodwork and apply more sophisticated statistical approaches (e.g., path models) that can strengthen the basis for causal relations. Though this study adds evidence of the gendered arrangement of housework using time-use diaries collected over a 1-week period in 2019, future research adopting a longitudinal design can reveal trends in household labor, which is particularly important given the societal changes that have taken place since the onset of the COVID-19 pandemic. Another fruitful direction for future research is to explore how arrangements of foodwork are related to coupled partners’ life cycles, professional categories, labor schedules, and other socio-demographic characteristics, which has not been examined in this study due to data limitations. While partnerships can take many forms, most of the research and the data collected in our study are limited to heterosexual couples and more research on other setups of cohabiting adults is needed.

## Figures and Tables

**Figure A1.**
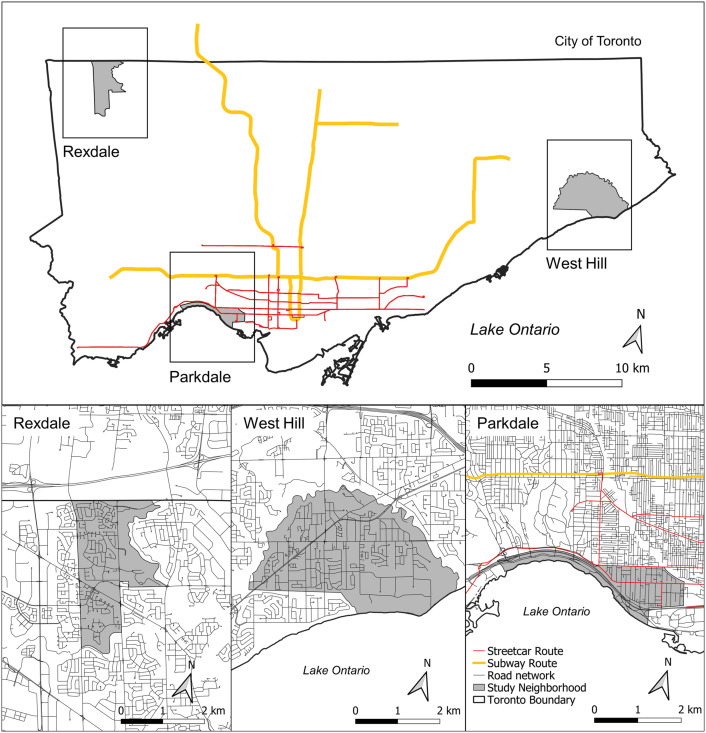
Maps of the City of Toronto (upper) and neighborhoods of Rexdale (bottom left), West Hill (bottom middle), and Parkdale (bottom right). Redrawn and adjusted based on Figure 1 in Smith et al., 2021 and Figure 2 in Liu et al., 2021. Boundaries of Rexdale, West Hill, and Parkdale, respectively correspond to the neighborhood boundaries of Mount Olive-Silverstone-Jamestown, West Hill, and South Parkdale where participants were recruited

## Appendix A. Descriptions of the three study neighborhoods

The three study neighborhoods were purposely chosen to have comparable low-to-moderate-income levels and contrasting densities of the built environment (Figure A1). Parkdale (Median household income: $41,761 in Canadian dollars, compared to the Toronto median of $65,829) located near downtown is densely populated (9,583 people per square kilometer). The other two neighborhoods, Rexdale (Median household income: $55,334) and West Hill (Median household income: $56,051) have a lower population density (7,291 and 2,856 people per square kilometer in Rexdale and West Hill, respectively) (City of Toronto, 2016). The population density of Rexdale was only slightly lower than Parkdale because Rexdale is characterized by a mix of houses and high-rise apartment buildings in a suburban context. In this study, Parkdale is categorized as an urban neighborhood, while the other two neighborhoods are regarded as suburban.
